# Post-COVID-19 fatigue: A systematic review

**DOI:** 10.3389/fpsyt.2022.947973

**Published:** 2022-08-11

**Authors:** Jian Joli, Patrizia Buck, Stephan Zipfel, Andreas Stengel

**Affiliations:** ^1^Department of Psychosomatic Medicine and Psychotherapy, University Hospital Tübingen, Tübingen, Germany; ^2^Charité Center for Internal Medicine and Dermatology, Department for Psychosomatic Medicine, Charité-Universitätsmedizin Berlin, Corporate Member of Freie Universität Berlin, Humboldt-Universität zu Berlin, and Berlin Institute of Health, Berlin, Germany

**Keywords:** brain fog, etiology, COVID-19, long-COVID, post-COVID fatigue, psychosomatic, symptoms, therapy

## Abstract

Fatigue is recognized as one of the most commonly presented long-term complaints in individuals previously infected with SARS-CoV-2. This systematic review was performed to describe symptoms, etiology, possible risk factors related to post-COVID-19 fatigue and the therapeutic approaches used for the treatment of post-COVID-19 fatigue. For the systematic literature search the databases PubMed, Web of Science, Cochrane Library, and PsycInfo were used. All articles that met the inclusion criteria were analyzed for demographics, clinical data and treatment. Included were studies which focused on an adult population (18–65 years old); elderly patients and patients with chronic somatic diseases which can also cause fatigue were excluded. We identified 2,851, screened 2,193 and finally included 20 studies with moderate to high methodological quality, encompassing 5,629 participants. Potential risk factors for post-COVID-19 fatigue were old age, female sex, severe clinical status in the acute phase of infection, a high number of comorbidities, and a prediagnosis of depression/anxiety. Lastly, a possible autoimmune etiology was suspected. Several treatment approaches have been tested mostly in small and uncontrolled studies so far: a Chinese herbal formulation improved breathlessness and fatigue. Moreover, molecular hydrogen (H_2_) inhalation had beneficial health effects in terms of improved physical (6-min walking test) and respiratory function in patients with post-COVID-19. Patients also noticed improvement in fatigue after undergoing hyperbaric oxygen therapy (HBOT) and enhanced external counterpulsation (EECP). Lastly. muscle strength and physical function were improved after undergoing an 8-weeks biweekly physical therapy course including aerobic training, strengthening exercises, diaphragmatic breathing techniques, and mindfulness training. However, larger and controlled studies e.g., investigating the effect of physical and / or psychotherapy for patients with post-COVID-19 fatigue are urgently warranted.

**Systematic Review Registration:**

Unique Identifier: CRD42022320676, https://www.crd.york.ac.uk/PROSPERO/.

## Introduction

Corona virus disease (COVID-19), caused by severe acute respiratory syndrome corona virus 2 (SARS-CoV-2), has led to a global pandemic. From the beginning of the pandemic until today (mid of May 2022), more than 519 million people worldwide have been infected with SARS-CoV-2 according to the world health organization, of whom more than 6 million have died. The corona virus has now spread to more than 190 countries. Currently, the highest numbers of cases are reported in the United States, Brazil, India, Turkey, and Russia. In Europe, Italy, Spain, France, Germany, and the United Kingdom have the highest number of corona virus infections.

It has become increasingly clear that infected patients have symptoms not only in the acute phase, but also after recovery from the initial infection (1). A recent meta-analysis including 4828 patients with post-COVID-19 showed that symptoms and post-acute sequelae of SARS-CoV-2 can persist weeks to months after the infection (2). These patients who reported persistent symptoms have been termed “long haulers” or described as having long COVID, post-acute COVID-19, persistent COVID-19 symptoms, post COVID-19 manifestations, long-term COVID-19 effects, post-acute sequelae of COVID-19 (PASC), or post-COVID-19 syndrome (3). Based on NICE guideline on long COVID (4); long COVID is defined as signs and symptoms that develop during or following an infection consistent with COVID-19 and which continue for more than 4 weeks. NICE recommends using the term post-COVID syndrome from 12 weeks after infection and when symptoms are not explained by an alternative diagnosis. Since in the 20 studies included in our systematic review the symptoms are reported on av-erage 20, 5 weeks after infection, we refer to the symptom(s) – according to the NICE guide-lines as Post-COVID fatigue.

The most prevalent symptoms reported by patients were: fatigue (64%), dyspnea (40%), depression/anxiety (38%), arthralgia (24,3%), headache (21%), and insomnia (20%) (2). Fatigue is a common and debilitating symptom in people with neurological (and oncological) disorders. Despite significant efforts to explain the pathogenic mechanisms of fatigue, current knowledge is limited. This may be because the cause of fatigue often cannot be attributed to a single source. Changes in neurotransmitter levels, inflammation, psychiatric disorders, psychosocial burden, cognitive dysfunction and substrate metabolism/availability are potential contributors to fatigue (5). Post-COVID-19 fatigue is defined as a decrease in physical and/or mental performance resulting from changes in central, psychological and/or peripheral factors resulting from COVID-19 disease (5).

In this systematic review we focused on persistent fatigue after acute COVID-19 infection, defined here as 2 weeks or greater post symptom onset. The review aimed to describe all symptoms related to post-COVID-19 fatigue, its possible etiology and risk factors as well as treatment approaches employed so far.

## Methods

### Data sources and searches

The protocol of this systematic review was registered on the International Prospective Register of Systematic Reviews (PROSPERO; registration number: CRD42022320676). The planning and conduct of this systematic review were carried out following the Cochrane guidelines for Systematic Reviews (6) and the Preferred Reporting Items of Systematic Reviews and Meta-Analyses (PRISMA) criteria catalog (7).

A systematic literature search developed by using the PICO model was conducted on April 14^rd^ 2022 on the following databases: PubMed/Medline, the Cochrane Library, PsycInfo, and Web of Science. There were no reservations related to the status, language or date of publication. The strategies developed and used for each database are presented in the Supplementary material. The following search terms were used: post OR post-infectious OR post-recovery OR postviral OR long OR long-term AND fatigue OR fatigue syndrome OR chronic fatigue AND coronavirus OR COVID-19 OR SARS-CoV-2.

Our search was based on a review question developed according to the PICO scheme i.e., Population (P): Patients, who have undergone a COVID-19 infection and suffer from post-COVID-19 symptoms, Intervention (I): all diagnostic tests (e.g., autonomic testing), interviews, self-reported fatigue tools / assessments / questionnaires, and therapy approaches used for patients with post-COVID-19, Comparison (C): patients, who had COVID-19 infection but no post-COVID-19 symptoms, and Outcome (O): all signs, symptoms, risk factors, pathophysiology related to fatigue after COVID-19 infection.

### Criteria for including studies

Original studies focusing on patients with post-COVID-19 fatigue were eligible for inclusion in this systematic review. Post-COVID-19 was initially defined as diagnosis when patients had at least one symptom beyond 2 weeks following acute infection (8). Studies with participants, who were in the acute phase during the study (< 2 weeks after being positive) were excluded. Since in the 20 studies included in our systematic review the symptoms are reported on average 20, 5 weeks after infection, we refer to the symptom(s) – according to the NICE guidelines as post-COVID fatigue.

Included were studies which focused on an adult population (older than 18). Those with data on geriatric patients over 65 years old (due to possible comorbidities in older age which can cause fatigue) and patients with chronic somatic diseases, which can also cause fatigue, were excluded. Only studies which reported cases with confirmed COVID-19 positive testing were included. Cases were defined as confirmed COVID-19 positive if they met one of the following criteria:

- Nasal, nasopharyngeal, oropharyngeal swab or nasotracheal, or blood samples tested positive for SARS-CoV-2 nucleic acid by using real-time reverse-transcriptase polymerase chain reaction assay (RT-PCR).
- A positive SARS-CoV-2 antibody (serology) test.

Studies were considered possibly eligible if they contained data from one or more patients encompassing randomized controlled trials, prospective cohort studies, cross-sectional studies and case reports. Non-original studies (meeting/conference/congress abstracts, notes and narrative reviews), animal studies, articles with non-topic-specific content, editorials, comments, hypotheses, opinions, dissertations, books or letters were excluded from further examination. Reviews, except for narrative reviews, were not generally excluded directly, but rather examined for potentially important primary sources if relevant to the topic. There were no restrictions related to the time of publication and time/duration of follow-up. Articles which met the criteria described above and were written in English were eligible for inclusion.

### Screening and full-text review

**Figure 1:**
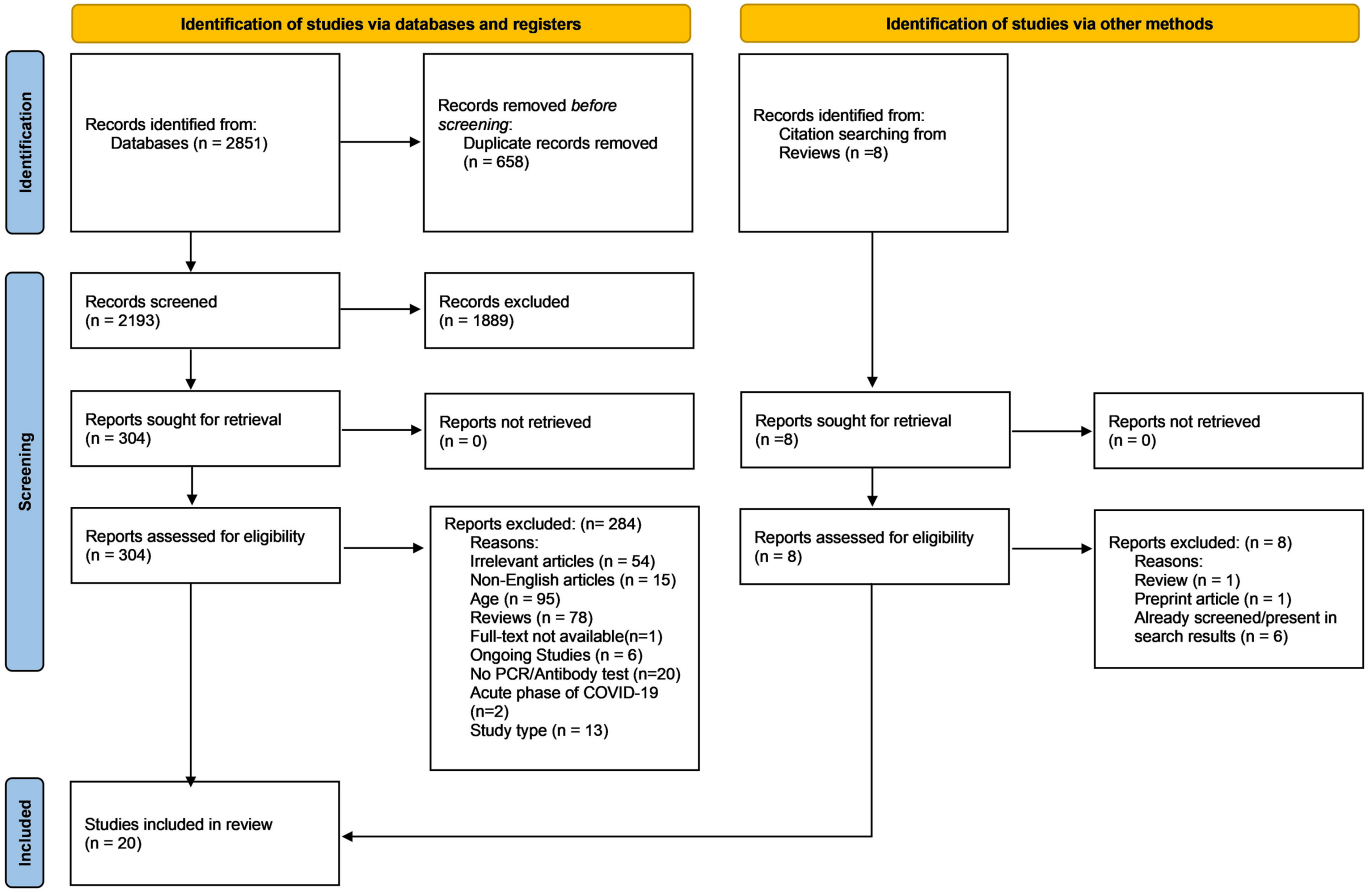
Prisma flow chart.

Before screening, duplicates were removed. This step was performed independently by two investigators (J.J. and P.B.) and the number of titles was compared afterwards. Then, titles and abstracts of identified studies retrieved using the search strategy were independently screened by two investigators (J.J. and P.B.) to identify studies that potentially meet the inclusion criteria mentioned above. The full texts of eligible studies were retrieved and independently assessed for eligibility by the two investigators. Any disagreement between them over the eligibility of particular studies was resolved through discussion with a third investigator (A.S.). The entire screening process is shown in the PRISMA flow diagram (Figure 1).

### Data extraction

The following information was extracted: Study data (author, year, country, study design, description of the included population with sample size and characteristics, duration of follow-up, ascertainment of COVID-19 and fatigue), demographic data (age and gender of included population, comorbidities), clinical data (symptoms and signs related to post-COVID-19), laboratory data (C-reactive protein, ferritin, antinuclear antibodies, D-dimer, lactate dehydrogenase, interleukin-6, white blood cells), etiology and possible risk factors (comorbidities, autoimmune diseases, gender, age), diagnostic tests (scales and questionnaires used for identifying fatigue after COVID-19 infection, chest-x-ray, brain MRI, spine MRI, electroencephalogram, autonomic testing, electrocardiogram, blood pressure, heart rate variability, electromyogram), and therapy approaches used for the treatment of post-COVID-19 fatigue.

### Assessing the methodological quality or risk of bias of included studies

The risk of bias assessment was performed on the premises of the study design as presented below:

- Randomized clinical trials: Cochrane risk of bias tool (6).
- Case-control study, non-randomized trials: ROBINS-I (9).
- Prevalence cross-sectional study: the Joanna Briggs Institute checklist for prevalence studies.
- Case reports: National Institute of Health quality assessment tool for case series studies.

Two independent investigators (J.J. and P.B.) performed the evaluation and a third investigator (A.S.) was involved in case of dissensus. We judged the quality of studies that used therapeutic interventions using questions such as: Was the intervention well defined? Were there any deviations from the planned intervention?

### Analysis of data

The prevalence of fatigue was estimated by dividing the number of patients with fatigue by the total number of patients with COVID-19 in the sample multiplied by 100 to estimate the percentage in each study.

We performed a narrative analysis (qualitative synthesis) to present the frequency of symptoms and signs related to post-COVID-19 fatigue.

## Results

### Search results

The search strategies retrieved 2,851 records, and 658 duplicates were removed initially. After reading of titles and abstracts (first phase), 1,889 references did not meet the inclusion criteria mentioned above and were excluded. The full texts of the 304 remaining records were read (second phase), 54 studies were excluded because they were not related to topic. Six ongoing studies were excluded. Other exclusion reasons were: non-English articles, age, and no confirmation of COVID-19 by RT-PCR or antibody test. Through the citation search from reviews, no additional studies were included. Finally, 20 studies were included in this systematic review. The PRISMA flow diagram is presented in Figure 1.

The 20 included studies encompass 5 studies that met the inclusion criteria but involved patients with comorbidities to compare this cohort with the fatigued cohort without pre-diagnoses. In one study participants were followed up after a mean duration of recovery from COVID-19 of only 12 days. However, these participants had 2 consecutive negative PCR tests before follow-up.

### Characteristics of the included studies

We present the results of the studies in Table 1 including: main characteristics of included studies including country, study design, description of the population, duration of follow-up and methods used for fatigue ascertainment.

**Table 1:** Characteristics of the included studies.

**Author, Year, Reference**	**Country**	**Study design**	**Population included**	**Sample size (*n*) and characteristics**	**Follow-up duration**	**Ascertainment of COVID-19**	**Ascertainment of fatigue**	**Results**	**Possible risk factors associated with persistent fatigue**
Nehme, 2021, (1)	Switzerland	Prospective longitudinal study	non-hospitalized subjects	*n* = 629 mean age: 42.1 years gender: 60.9% F, 39.1% M	Baseline (acute phase), 30–45 days follow up and 7–9 months follow-up	RT-PCR	ECOG (29) performance scale	Fatigue was reported at: - Baseline: 61.7% - 30–45 days follow-up: 17.9% - 7–9 months follow-up: 20.7% additional symptoms such as myalgia, difficulty concentrating, and memory loss were reported	female and older patients had a higher risk suffering from fatigue
Graham, et al. (10)	United States	prospective case-control study	non-hospitalized post-COVID-19 patients; at least 6 weeks after symptom onset and those who had neurological symptoms lasting over than 6 weeks. negative patients meeting IDSA (30) guidelines were included as a comparison group	*n* = 100 mean age: 43.2 years gender: 70% F, 30% M	follow-up time: mean 5.27 months from onset	RT-PCR or SARS-CoV-2 antibody testing	PROMIS assessment (31), NIH toolbox (30, 32) Blood laboratory testing, brain MRI, MR vessel wall imaging, spine MRI, EMG and EEG	85% reported fatigue, 81% Brain fog, 32% short-term memory deficit, 27% attention deficit, and 33% reported insomnia no different results in PROMIS and NIH or in laboratory tests between the two groups	possible autoimmune etiology of post-Covid premorbid depression/anxiety suggesting a possible neuropsychiatric vulnerability to becoming a “long hauler” after SARS-CoV-2 infection
Elanwar, et al. (11)	Egypt	case-control study	COVID-19 long-haulers who were diagnosed as having PIFS (33) and another sample of age- and sex-matched volunteers (recovered from COVID-19 without any residuals)	*n* = 92 mean age: 54.5 years in fatigue group and 51 years in non-fatigue group gender: 23.9% M, 76.1% F in fatigue group and 41% M and 58.7% F in non-fatigue group	cross-sectional after recovery; recovery was defined as having 2 negative tests	not reported	fatigue questionnaire (34) to assess physical and mental fatigue motor and sensory nerve-conduction studies to exclude any neuropathy electromyography examinations to exclude any myopathy	79.3% reported fatigue median value for fatigue was 6 patient in PIFS had longer disease duration and higher levels of CRP and ferritin.	high levels of ferritin during acute phase and duration of COVID-19 illness
Kamal, 2021 (12)	Egypt	retrospective cross-sectional study	survivors from COVID-19 from general population	*n* = 287 mean age: 32.3 years gender: 64.1% F, 35.9% M	cross-sectional	not reported	questionnaire ad *hoc*	72.8% reported fatigue 38% reported anxiety 28,6% reported depression 28.6% reported dementia	severity of COVID-19 was related to the severity of post-COVID-19 manifestations
Ganesh, et al. (13)	United States	retrospective cross-sectional study	patients identified by history of positive PCR for SARS-CoV-2 who meet the clinical definition of recovery from COVID-19 (resolution of symptoms or >30 days post initial positive PCR test)	*n* = 817 mean age: 44 years gender: 61.1% F, 38.9% M	Cross-sectional, the mean interval between being positive and survey response was 68.4 days	RT-PCR	PROMIS (35) scales to assess physical and social dysfunction PROMIS domains were: fatigue, social roles, pain, physical functions and sleep	dysfunction was reported in following domains: ability to participate in social roles (43.2%), pain (17.8%), and fatigue (16.2%)	female gender has a higher risk of fatigue
El Sayed, 2021 (14)	Saudi Arabia	Cross-sectional observational study with longitudinal component	patients with COVID-19 after 2 consecutive negative PCR tests who attended the pulmonology clinic for follow up and psychiatric department for assessment	*n* = 200 mean age: 36.6 years gender: 43% F, 57% M	cross-sectional after recovery	RT-PCR	fatigue assessment scale (36)	Mean fatigue score was 40.8, which means a very high level of fatigue (highest level=50) positive correlation between fatigue and anhedonia negative correlation between duration after recovery and fatigue	Not assessed
Townsend, et al. (15)	Ireland	case-control study	post-COVID-19 patients with and without Vitamin D supplementation	*n* = 149 mean age: 48 years gender: 59% F, 41% M	median time for follow-up was 79 days from initial infection	RT-PCR	Chalder-fatigue score	a positive association between fatigue scores and higher vitamin D levels	Not assessed
Townsend, et al. (16)	Ireland	observational single center study	Post-COVID-19 patients; at least 6 weeks after: - date of last acute COVID-19 symptoms (for outpatients) - date of discharge for those who were admitted during acute phase	*n* = 128 mean age: 49.5 years gender: 53.9% F, 46.1% M	56 days to 12 weeks after initial diagnosis	RT-PCR	CFQ-11 (34, 37) blood sampling to assess any relationship between fatigue and pro-inflammatory cytokines	52.3% reported fatigue no relationship between fatigue and pro-inflammatory cytokines (absence of a specific immune signature associated with persistent fatigue)	female patients and patients with a history of depression/anxiety or anti-depressant use had higher risk for suffering from fatigue
Townsend, et al. (17)	Ireland	case-control study	20 patients with post-COVID-19 patients and fatigue and 20 patients with post-COVID-19 without fatigued	*n* = 40 median age: 44.5 years gender: 36% F, 64% M	median time to follow up: 166.5 days	RT-PCR	CFQ-11 Autonomic testing: Ewing's autonomic function test battery (38), continuous blood pressure, electrocardiogram and heart rate variability	no differences between fatigued and non-fatigued patients on autonomic-testing or on 24-h blood pressure 70% of the fatigued cohort reported orthostatic intolerance at time of active standing fatigue was associated with increased anxiety, with no patients having a pre-existing diagnosis of anxiety	Not assessed
Margalit, et al. (18)	Israel	case-control study	previously healthy post-COVID-19 patients, at least 2 months after initial infection	*n* = 141 mean age: 47 years gender: 59% F, 41% M	212–240 days after initial infection	RT-PCR	Self-reported on a scale from 0 (not present) to 3 (severe)	46.8% reported fatigue	having more children and a lower proportion of hypothyroidism was associated with fatigue in patients with post-COVID-19
Dayrit, 2021, (19)	USA	case report	38-years-old Hispanic woman with no preexisting health conditions	*n* = 1 age: 38 years gender: 100% F	patient underwent treatment after 3 months of her persistent symptom onset	RT-PCR	*ad hoc*	fatigue and brain fog brain fog and fatigue were improved after treatment with EECP	Not assessed
Mayer, et al. (20)	USA	case report	37-years-old non-hospitalized woman	*n* = 1 age: 37 years gender: 100% F	64–120 days after initial diagnosis	RT-PCR	*ad hoc*	fatigue and cognitive fog were reported, which had not improved after physical therapy	Not assessed
Bhayiat, et al. (21)	Israel	case report	55-years-old previously healthy man	*n* = 1 age: 44 years gender: 100% M	patient underwent the therapy after 3 months of acute Infection	RT-PCR	*ad hoc*	fatigue, memory problems, worsening of multitasking abilities, low energy, breathlessness, and reduced physical fitness after 15 sessions of HBOT he noted less fatigue and an improvement in his previously low energy	Not assessed
Pang, et al. (22)	China	case-control study	Patients with post-COVID-19 after two consecutive negative results of RT-PCR tests with at least a 1-day interval between tests	*n* = 388 mean age: 46 years gender: 62% F, 38% M	Baseline, 7-days and 14-days follow-up after inclusion and randomization	RT-PCR	Borg scale (39)	improvement of fatigue after 7 days no adverse event related to QJYQ was recorded	Not assessed
Townsend, et al. (23)	Ireland	cross-sectional study	hospitalized (including ICU) and not-hospitalized patients with post-COVID-19	*n* = 153 median age: 48 years gender: 57.5% F, 42.5% M	61–117 days after diagnosis	RT-PCR	CFQ-11 chest X-ray, blood sampling, 6MWT (40) and modified Borg dyspnea scale (MBS) (41) to find associated factors with persistent poor health after COVID-19	48% met the case definition for fatigue and this was not associated with the severity of initial infection or abnormal chest x-ray but associated with an increased MBS score 62% did not return to full health which was associated with an increased MBS score	Not assessed
Augustin, et al. (24)	Germany	prospective and longitudinal study	asymptomatic patients, who had previously positive PCR; at least 6 weeks after symptom onset or positive PCR	*n* = 353 median age: 43 years gender: 53.3% F, 46.5% M	6–8 months following onset of symptoms	RT-PCR	questionnaire *ad hoc*	14.7% reported fatigue	female patients and individuals with a prior diagnosis of depression or anxiety had a higher risk of suffering from fatigue
Fernandez-de-la-penas, 2021, (25)	Spain	retrospective study	hospitalized patients	*n* = 1950 mean age: 61 gender: 46.9% F, 53.1% M	Mean 11.2 ± 0.5 months after hospital discharge	RT-PCR and radiological finding	self-reported *via* structured telephone interview conducted by trained healthcare professionals	61.4% reported fatigue 16% (6/38) reported being no longer able to participate in a sport or recreational activity because of their ongoing symptoms	Not assessed
Vanichkachorn, et al. (26)	USA	Observational study	patients with post-Covid-19; at least after 4 weeks from being positive or symptomatic start.	*n* = 100 mean age: 45.4 years gender: 68% F, 32% M	mean time of presentation was 93.4 days after diagnosis	RT-PCR or antibody (serology) test	function focused interview	80% reported fatigue, 59% reported neurological complaints such as cognitive impairment and sleep disturbance	Not assessed
Botek, et al. (27)	Czech Republic	randomized, single-blind, placebo-controlled study	Non-vaccinated post-COVID-19 patients	*n* = 50 mean age: 38 years F, 44 years M gender: 48% F, 52% M	21–33 days after initial infection	RT-PCR	self-reported on a 5-point scale (0 = none, 4 = severe)	80% reported fatigue molecular hydrogen (H2) inhalation had beneficial health effects in terms of improved physical (6MWT) and respiratory function in patients with post-COVID-19 no significant improvement in fatigue between the two groups	Not assessed
Schaeffer, et al. (28)	Canada	case-control study	patients with post-COVID-19, 3 months after discharge or last positive SARS-CoV-2 test	*n* = 49 mean age: 47 years gender: 47% F, 53% M	Cross-sectional, 3 months after infection	RT-PCR	questionnaire *ad hoc*	higher depression and anxiety scale in patients with post-COVID-19 fatigue in comparison to the control group	Not assessed

*6MWT, 6-Minute walk test; CFQ-11, chalder fatigue scale; COVID-19, Coronavirus disease 2019; CPET, Cardiopulmonary exercise test, ECOG, the eastern cooperative oncology group; EECP, enhanced external counterpulsation; F, Female; HBOT, hyperbaric oxygen therapy; ICU, Intensive care unit; IDSA, Infectious Diseases Society of America Guidelines; M, Male; MBS, Modified Borg scale; MRI, magnetic resonance imaging; n, Number; NIH, national institute of health; PIFS, Postinfectious fatigue syndrome; PROMIS, patient-reported outcome measurement information system; QJYQ, QingjinYiqi granules; RT-PCR, reverse transcription polymerase chain reaction; SARS-CoV-2, Severe acute respiratory syndrome coronavirus 2*.

The included studies comprised a total sample of 5,629 participants (aged 18 to 65 years). Of the 20 included studies, 3 were prospective cohort studies, 2 were retrospective cohort studies, 3 were cross-sectional studies, 6 were case-control studies, 1 was a randomized single-blind, placebo-controlled study, 2 were observational studies and 3 were case reports. Five studies analyzed data from the US, 2 from Egypt, 4 from Ireland, 2 from Israel, 1 from China, and 1 each analyzed data from Saudi Arabia, Czech Republic, Switzerland, Germany, Canada, and Spain. Study populations ranged from 40 to 1,950 participants; except for the three case reports which analyzed just one patient. The median or mean follow-up periods ranged from 1 to 9 months.

### Risk of bias of included studies

The risk of bias was categorized into low risk, some concerns, and high risk. The included studies were evaluated regarding five various forms of bias:

- Bias in selection of participants for the study.
- Bias due to deviations from intended interventions.
- Bias due to missing data.
- Bias in measurement of the outcomes.
- Bias in selection of the reported results.

Ten studies were classified as presenting high quality (low risk), and the remaining 10 as moderate quality (some concerns) data. The reasons for these concerns were as follows: the outcomes were measured *via* questionnaires or were self-reported by the participants (domain 4: bias in measurement of outcomes). We present the judgments for the bias in Table 2.

**Table 2:** Risk of bias assessment.

**References**	**Selection of participants**	**Deviations from interventions**	**Missing data**	**Measurment of outcomes**	**Selection of reportd results**	**Total**
(1)	□	□	□	□	□	□
(10)	□	□	□	□	□	□
(11)	□	□	□	□	□	□
(12)	□	□	□	□	□	□
(13)	□	□	□	□	□	□
(14)	□	□	□	□	□	□
(15)	□	□	□	□	□	□
(16)	□	□	□	□	□	□
(17)	□	□	□	□	□	□
(18)	□	□	□	□	□	□
(19)	□	□	□	□	□	□
(20)	□	□	□	□	□	□
(21)	□	□	□	□	□	□
(22)	□	□	□	□	□	□
(23)	□	□	□	□	□	□
(24)	□	□	□	□	□	□
(25)	□	□	□	□	□	□
(26)	□	□	□	□	□	□
(27)	□	□	□	□	□	□
(28)	□	□	□	□	□	□

*
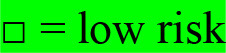
; 
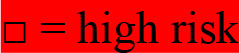
; 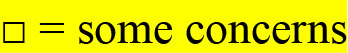
*.

### Results from the included studies

#### Scales and definition criteria used for identifying post-COVID-19 fatigue

Elanwar et al. (11) defined participants as patients with post-COVID-19 if they were diagnosed with postinfectious fatigue syndrome (PIFS). In order to fulfill the definition of PIFS, patients had to have persistent fatigue for at least 6 months after recovery. Post-COVID-19 fatigue was defined in the other studies by persistent fatigue symptoms at least 6 weeks after recovery or after being negative using RT-PCR test. Kamal et al. (12) did not explain exactly how they defined fatigued patients. In the studies of Ganesh et al. (13) and Graham et al. (10), fatigue domains were assessed using patient-reported outcome measurement information system (PROMIS) with a 5-point Likert scale ranging from 1 = never to 5 = always. El Sayed et al. (14) used the fatigue assessment scale. Elanwar et al. (11), Townsend et al. (15–17) used the Chalder fatigue scale providing a total fatigue score from 0-4. Scores of 2 or above are regarded as fatigued.

All of the scales mentioned above consist of items that measure both the experience of physical and/or mental fatigue and the interference of fatigue on daily activities over the past weeks. Examples of items are: “How often did you feel tired?”, “How often were you too tired to take a bath/shower?”, “I get tired very quickly”, “I don't do much during the day”, “Physically, I feel exhausted”, “I have problems thinking clearly”, and “Mentally, I feel exhausted”.

In the study of Pang et al. (22) the patients used the Borg scale (39) to grade the level of their fatigue. This is a scale ranging from 0 = nothing at all to 20 = very, very severe (maximal).

#### Signs and symptoms related to fatigue experienced by participants

**Figure 2:**
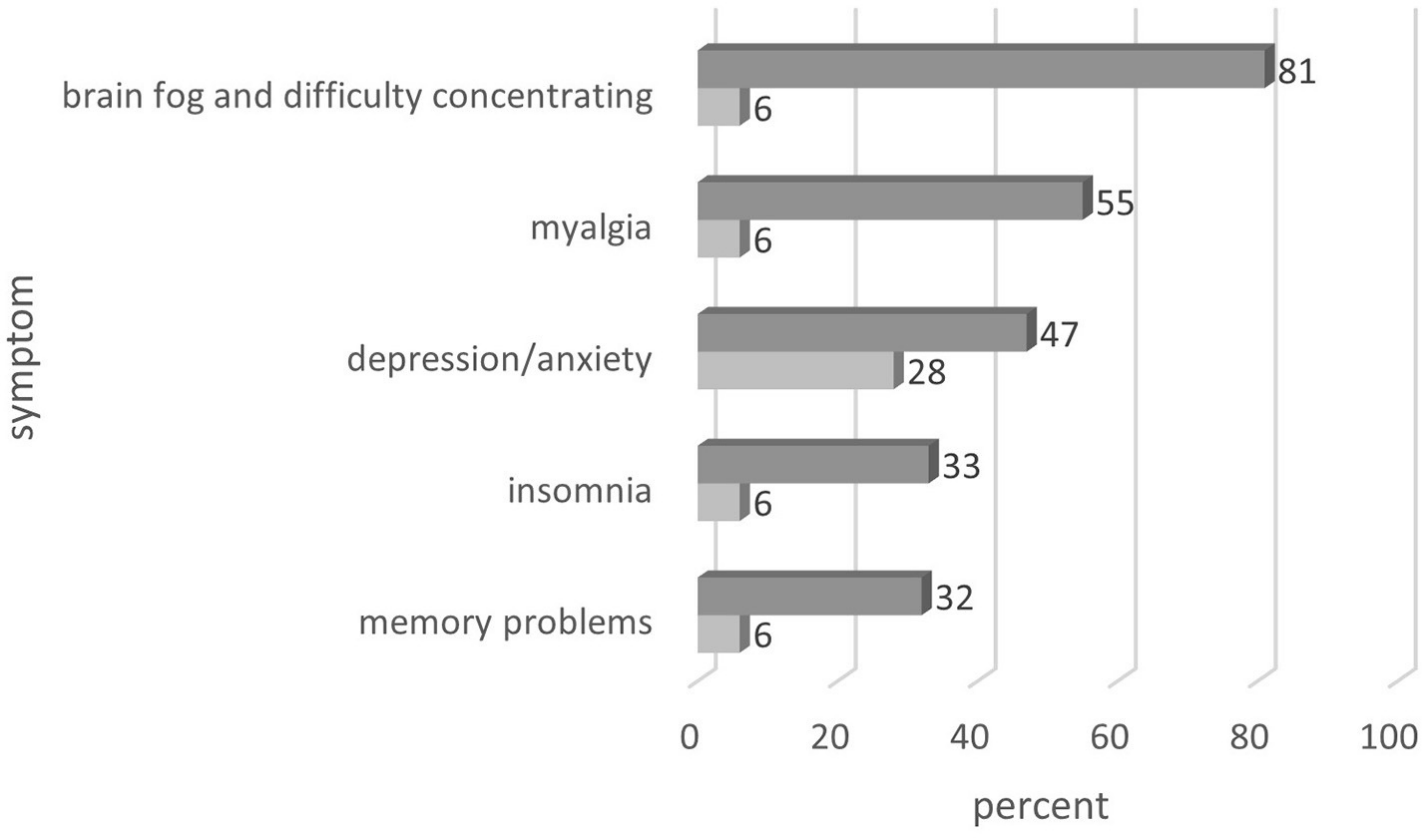
Additional symptoms experienced by patients with post-COVID-19 fatigue (Frequency: from %—to %).

The participants reported several symptoms related to fatigue in different proportions. Fatigue (by definition) was always present, anhedonia, brain fog and difficulty concentrating (up to 81%), myalgia (up to 55%), depression/anxiety (up to 47%), insomnia and sleep disturbance (up to 33%), and dementia or loss of memory (up to 32%). A summary of symptoms reported in the included studies is presented in Figure 2.

#### Etiology and risk factors

A total of 7 studies reported aspects that were investigated as potential risk factors for post-COVID-19 fatigue. Kamal (12), Nehme (1), and Townsend (16) described that older and female patients have a higher risk of suffering from fatigue after COVID-19 infection. Kamal (12), El Sayed (14), and Elanwar (11) showed that the longer the duration of the acute phase (or of the disease recovery) and the more severe the disease, the higher the risk of suffering from fatigue. Finally, patients with comorbidities were more affected to have persistent symptoms (12).

Similar to these results, Graham (10) suggested in their study a possible neuropsychiatric vulnerability to becoming long haulers after COVID-19 infection because premorbid depression/anxiety was prevalent in their cohort. Lastly, Townsend (16) found a significant association between pre-existing depression diagnosis and antidepressant use and subsequent development of severe fatigue.

**Figure 3:**
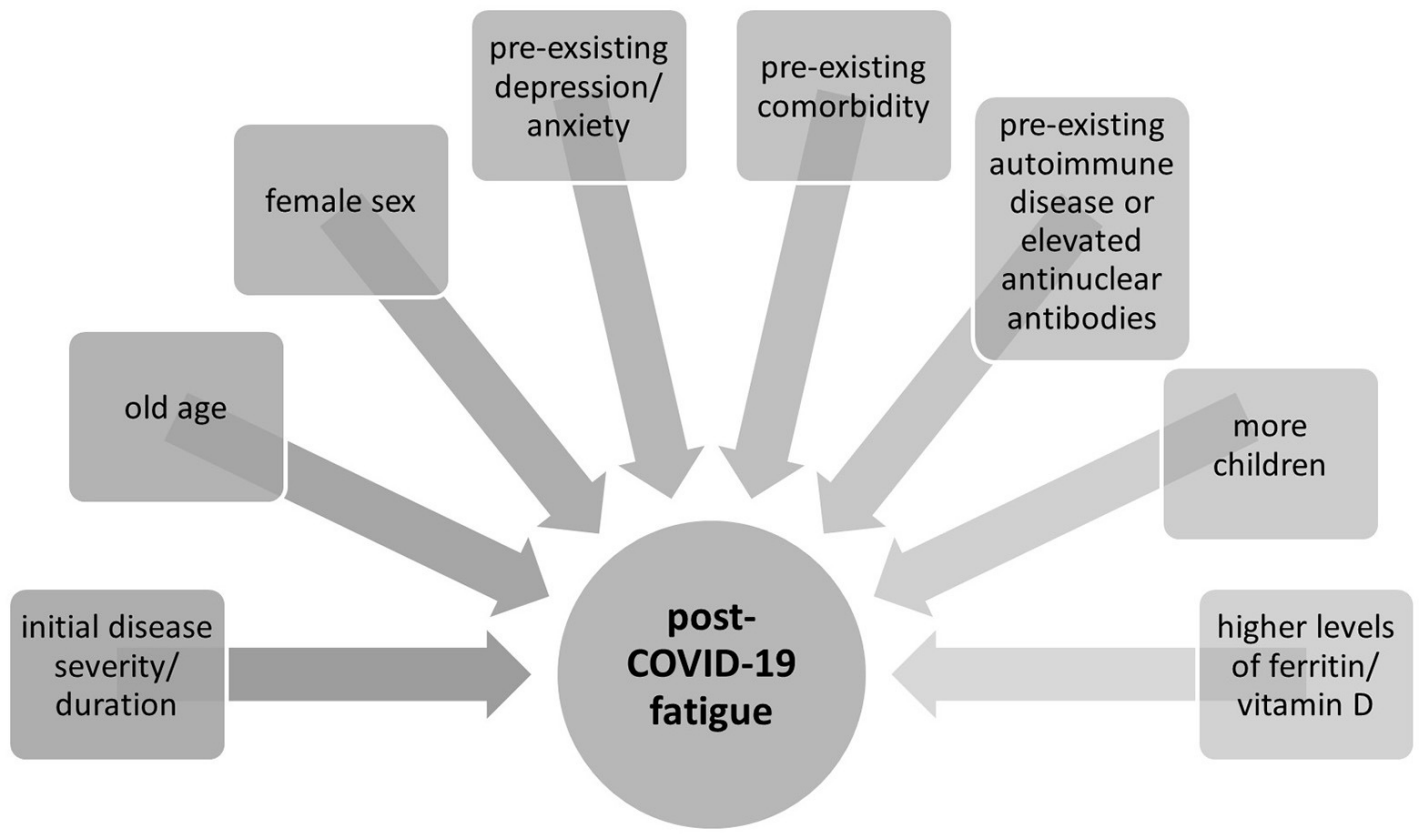
Etiology and risk factors of post-COVID-19 fatigue.

Graham (10) showed that the prevalence of preexisting autoimmune disease and elevated ANA (antinuclear antibodies) in the cohort of participants with post-COVID-19 compared to the general population possibly pointing toward an autoimmune contribution (10). In addition, Ganesh (13) hypothesizes that sex differences in the immune response to COVID-19 may be related to the development of persistent symptoms after COVID-19 infection. Elanwar (11) found higher levels of ferritin in patients with post-COVID-19 fatigue compared to the control group without fatigue. Margalit et al. (18) reported in their study that patients with post-COVID-19 fatigue had more children and lower proportion of hypothyroidism. Lastly, Townsend et al. (15) showed a positive association between fatigue scores and higher vitamin D levels in their cohort. A summary of associated risk factors reported in included studies is presented in Figure 3.

#### Therapeutic approaches

Dayrit et al. (19) presented a case of a 38-year-old female patient who experienced post-COVID-19 sequelae, including fatigue, headache, shortness of breath, and brain fog for 3 months before she underwent enhanced external counterpulsation (EECP) of 1-h sessions, three times per week for 5 weeks. EECP is a non-invasive therapy for patients with chronic stable angina and/or heart failure of ischemic etiology. Standard therapy involves 35 1-h treatments over seven weeks. The patient lies on a treatment table with compression cuffs securely wrapped around the calves, thighs and buttocks. These cuffs induce a sequence of drainage from distal to proximal simultaneously before the onset of diastole and systole. Inflation and deflation are tailored specifically to the patient's ECG to optimize treatment use. Compression produces pulsating shear stress, this is the same as adjusting Vasodilators (e.g., nitric oxide) and proinflammatory agents (e.g., tumor necrosis factor alpha). This leads to physiological and biochemical changes in the vasculature with the aim to stimulate angiogenesis and increase coronary function (42). Dayrit et al. are the first to use EECP for persistent post-COVID-19 symptoms. After 1 week of treatment, the patient's brain fog improved. Shortness of breathing improved after 1.5 weeks and the patient reported returning to pre-COVID-19 health and fitness after approximately 5 weeks of EECP treatment. However, this was only one case and a control person was lacking.

Mayer et al. (20) presented another case of a 37-old-year woman who experienced persistent symptoms after COVID-19 infection including dyspnea, headache and cognitive fog. She previously participated in an outpatient physical therapist evaluation, which showed deficits in exercise capacity, reaching 50% of the expected 6-min walk distance for her age (6MWD). Moreover, she had minor reductions in muscle strength and cognitive function. She underwent a physical therapy examination that was based on the guidelines for intensive care unit (ICU) survivors as well as the COVID-19 core outcome measure guidelines from the academies and sections of the American physical therapy association. Subsequently, she participated in an 8-week biweekly physical therapy course that included aerobic training, strengthening exercises, diaphragmatic breathing techniques and mindfulness training. Metabolic equivalent (METS) values for tasks increased as the program progressed. The patient's muscle strength, physical performance, and physical function improved. The 6MWD increased by 199 m, which is 80% of the predicted distance for her age. However, self-reported quality of life (QoL) scores did not improve. After physical therapy, the patient continued to have migraine, dyspnea, fatigue, and cognitive impairment. Also in this case-report (*n* = 1) there was no control group.

Bhaiyat et al. (21) reported in a case-report of a 55-year-old healthy man the use of hyperbaric oxygen therapy. The man suffered from persistent symptoms including fatigue, memory problems, low energy, breathlessness and reduced physical fitness, which all started 3 months after the acute infection of COVID-19. He underwent a hyperbaric oxygen therapy that included 60 sessions, 5 days per week, and each session encompassed exposure to 90 min of 100% oxygen at 2 atmosphere absolute with 5-min air breaks every 20 min. The patient noted less fatigue and an improvement in his previous low energy after 15 sessions. Additionally, he reported that his memory and multitasking ability returned to his pre-COVID-19 levels. The baseline brain MRI, prior to therapy showed a global decrease in the brain perfusion which was increased after therapy. However, this was only one case and a control person was lacking.

Pang et al. (22) used a drug called Qingjin Yiqi granules (QJYQ) for 388 patients with post-COVID-19 fatigue for 14 days. QJYQ contains 16 herbs. These herbs are initially extracted with water, followed by concentration and spray-drying to powder, after which excipients are added with the final mixture pelletized by dry granulation method. Each package contains 10 g, equivalent to 52 g of the crude drug. QJYQ has been recommended by the rehabilitation guidelines of integrated medicine for patients with post-COVID-19 in China (43, 44). In this study, data showed the QJYQ-group was superior to the control group in Borg scale, which was employed to evaluate perceived exertion and fatigue. Improvement in breathlessness and fatigue has been shown. No adverse event related to QJYQ was recorded.

Botek et al. (27) used in their randomized, single-blind, placebo-controlled study molecular hydrogen (H_2_) as a therapeutic gas for 50 patients with post-COVID-19 with a follow-up of 33 days because it has antioxidative, anti-inflammatory, anti-apoptotic and anti-fatigue properties (45–47). The protocol consisted of H_2_/placebo inhalation, 2 × 60 min/day for 14 days. Results showed that H_2_ therapy increased 6-min walking distance and improved forced vital capacity (FVC) and forced expiratory volume (FEV1) compared with placebo. These data suggested that H_2_ inhalation may have beneficial health effects in terms of improved physical and respiratory function in patients with post-COVID-19.

## Discussion

Fatigue was reported as one of the most common persistent symptoms in individuals infected with SARS-CoV-2 before. Persistent fatigue lasting at least 6 months is termed chronic fatigue syndrome (33). This may be observed after several viral and bacterial infections (48). In the included studies of the present systematic review, fatigue was a symptom that either occurred already in the acute phase or developed after recovery from the acute phase of infection. The included studies recruited patients for a maximum of 9 months, with fatigue as persistent symptom. This should be followed up for a longer period to see how long fatigue could persist after the acute infection of COVID-19.

The origin of fatigue can be—based on our current knowledge—explained by a biopsychosocial model of the disease (49). It can be caused by a variety of biological or physical dysfunctions (e.g., genetic factors). In addition, other factors may contribute to the development of fatigue in patients with post-COVID-19 such as cytokines released by SARS-CoV-2 infection that impair psychological defense mechanisms. Also, the prevalence of preexisting autoimmune disease and elevated ANA (antinuclear antibodies) in a cohort of participants with post-COVID-19 compared to the general population suggests the possibility of an autoimmune contribution (10). Lastly, Townsend et al. (17) clearly demonstrated the absence of significant dysautonomia in post-COVID-19 fatigue. Social factors (e.g., socioeconomic status) as well as psychological factors (e.g., emotional stress) can contribute individually or in combination to the development of fatigue (49). Psychological and social factors include experiences of helplessness in illness, avoidance behaviors, financial worries due to unemployment, and loneliness due to limitations in social contacts (49). However, the individual handling of fatigue (motivational factors, coping behavior, sleep habits) also represents an important factor. In our systematic review, a possible link between premorbid depression/anxiety and post-COVID-19 fatigue has been shown (16, 24). Taken, together, the most predisposing factors of persistent symptoms observed in patients with post-COVID-19 syndrome were old age, female sex, severe clinical status at acute phase, high number of comorbidities, premorbid depression/anxiety, hospital admission and oxygen supplementation at the acute phase (50).

Several non-mutually exclusive hypotheses regarding the pathogenesis of COVID-19 suggest that anti-inflammatory drugs may be beneficial for selected patients (51). Also psychotropic drugs (e.g., selective serotonin reuptake inhibitors) (52) can modulate pro-inflammatory cytokine levels, and may have beneficial effects on mood and cognition in COVID-19 survivors (53). However, data are lacking and the effect of these drugs on patients with post-COVID-19 fatigue should be investigated.

Based on the suggested vascular pathophysiology possibly contributing to post-COVID-19 fatigue (54), EECP may be an appropriate treatment for such patients. EECP is a none-invasive treatment for patients with chronic stable angina and/or ischemic heart failure (42). It has also been shown to enhance cerebral blood flow, collateralization in the ischemic regions of the brain, and cognitive function. Improving of the patient's symptoms in the case-report of Dayrit et al. (19) gives further rise to the hypothesis that post-COVID-19 fatigue may be related to intracerebral hypoperfusion possibly due to COVID-19 associated microemboli (55).

Based on our findings, rehabilitation programs like pulmonary rehabilitation using hyperbaric oxygen therapy or physical therapy including aerobic training, strengthening exercises, diaphragmatic breathing techniques as well as mindfulness training, might represent treatment options in patients with persistent symptoms after COVID-19. Also, a molecular hydrogen (H_2_) inhalation had beneficial health effects in terms of improved physical (6-min walking test) and respiratory function in patients with post-COVID-19 (27). Other therapeutic options are likely to appear as well and may have been missed here due to the focus on English written literature only. It would be also important to investigate whether (additional) psychotherapeutic approaches such as cognitive behavioral therapy, shown before to be beneficial for patients with long-lasting fatigue after Q-fever (affecting up to 30% of patients after the largest reported outbreak of Q-fever) could be an effective treatment for post-COVID-19 as also suggested by Vink et al. (56), however, actual data are lacking so far. Since most patients with post-COVID-19 fatigue also suffer from brain fog, myalgia, and depression/anxiety, more research including physical and psychological therapy is necessary in order to identify treatment options and ultimately improve the quality of life of patients with post-COVID-19 fatigue.

## Data availability statement

The original contributions presented in the study are included in the article/Supplementary material, further inquiries can be directed to the corresponding author.

## Author contributions

JJ and PB: performed the systematic search. JJ: wrote the first draft of the manuscript. AS: planned the study and together with SZ gave critical input throughout the study. All authors finalized the manuscript. All authors contributed to the article and approved the submitted version.

## Funding

Funding by Tübingen University Hospital, Department of Psychosomatic medicine and psychotherapy, and open access publishing fund of Tübingen University Hospital and of DFG(Deutsche Forschungsgemeinschaft).

## Conflict of interest

The authors declare that the research was conducted in the absence of any commercial or financial relationships that could be construed as a potential conflict of interest.

## Publisher's note

All claims expressed in this article are solely those of the authors and do not necessarily represent those of their affiliated organizations, or those of the publisher, the editors and the reviewers. Any product that may be evaluated in this article, or claim that may be made by its manufacturer, is not guaranteed or endorsed by the publisher.
