# Supplementary material for: Post-COVID-19 fatigue: A systematic review
Source: Front Psychiatry. 2022 Aug 11;13:947973. doi: 10.3389/fpsyt.2022.947973 (PMC9403611; doi:10.3389/fpsyt.2022.947973)
Supplement: Supplementary file 1 [file Data_Sheet_1.PDF]

07.07.2021 and (14.04.2022)

PUBMED

Search number	Query	Sort By	Filters	Results	Time
11	(#9) AND (#10)			394 (658)	02:37:28
10	(#2) OR (#5)			114,496	02:36:59
9	(#3) AND (#8)			13,852	02:36:10
8	(#1) OR (#4)			166,072	02:35:26
7	((post[MeSH Terms]) OR (post-infectious[MeSH Terms])) OR (post-recovery[MeSH Terms])			0	02:34:04
6	((post[MeSH Terms]) OR (post-infectious[MeSH Terms])) OR (post-recovery[MeSH Terms]) - Schema: all			0	02:34:04
5	(("Fatigue"[Mesh]) OR "Fatigue Syndrome, Chronic"[Mesh]) OR ("Fatigue Syndrome, Chronic/complications"[Mesh] OR "Fatigue Syndrome, Chronic/etiology"[Mesh] OR "Fatigue Syndrome, Chronic/physiopathology"[Mesh])	Most Recent		37,663	02:29:52
4	(((("Coronavirus"[Mesh]) OR "COVID-19"[Mesh]) OR ("COVID-19/complications"[Mesh] OR "COVID-19/physiopathology"[Mesh])) OR "SARS-CoV-2"[Mesh]	Most Recent		102,462	02:26:46
3	((((post[Title/Abstract]) OR (long[Title/Abstract])) OR (long-term[Title/Abstract])) OR (post-infectious[Title/Abstract])) OR (post-recovery[Title/Abstract])			2,410,649	02:22:17
2	(((((fatigue[Title/Abstract]))) OR (fatigue syndrome[Title/Abstract])) OR (postviral fatigue[Title/Abstract])) OR (chronic fatigue[Title/Abstract])			104,532	02:18:48
1	((Coronavirus[Title/Abstract]) OR (COVID-19[Title/Abstract])) OR (SARS-COV-2[Title/Abstract])			156,876	02:12:11

Cochrane Library

Search Results

#1 (Coronavirus):ti,ab,kw 3574

#2 (COVID-19):ti,ab,kw 5915

#3 (SARS-CoV-2):ti,ab,kw 255

#4 #1 OR #2 OR #3 6263

#5 MeSH descriptor: [Coronavirus] this term only 4

#6 MeSH descriptor: [COVID-19] this term only 467

#7 MeSH descriptor: [SARS-CoV-2] this term only 343

#8 #5 OR #6 OR #7 470

#9 #4 OR #8 6263

#10 (long OR long-term OR post OR chronic OR post-infectious OR post-recovery):ti,ab,kw 423607

#11 #9 AND #10 1333

#12 MeSH descriptor: [Fatigue Syndrome, Chronic] this term only 399

#13 ("fatigue disease"):ti,ab,kw 30

#14 ("postviral fatigue syndrome"):ti,ab,kw 12

#15 ("chronic fatigue syndrome"):ti,ab,kw 937

#16 (fatigue):ti,ab,kw 35771

#17 MeSH descriptor: [Fatigue] explode all trees 3906

#18 #12 OR #13 OR #15 OR #16 OR #17 35771

#19 #11 AND #18 99 (50)

Suche #19

Datenbanken	Anzahl der Treffer
Cochrane Reviews	1
Cochrane Protocols	0
Trials	98
Editorials	0
Special collections	0
Clinical Answers	0

Web of Science

Search number	Results	
#7	768 (234)	#6 AND #5<br>*Databases= WOS, BCI, BIOSIS, CCC, DRCI, DIIDW, KJD, MEDLINE, RSCI, SCIELO, ZOOREC Timespan=All years Search language=Auto*
#6	746,828	TS=(Fatigue OR Fatigue-Syndrome OR chronic-Fatigue OR postviral-Fatigue)<br>*Databases= WOS, BCI, BIOSIS, CCC, DRCI, DIIDW, KJD, MEDLINE, RSCI, SCIELO, ZOOREC Timespan=All years Search language=Auto*
#5	33,290	#4 AND #3<br>*Databases= WOS, BCI, BIOSIS, CCC, DRCI, DIIDW, KJD, MEDLINE, RSCI, SCIELO, ZOOREC Timespan=All years Search language=Auto*
#4	11,559,709	TS=(post OR long OR Chronic OR post-infectious OR post-recovery)<br>*Databases= WOS, BCI, BIOSIS, CCC, DRCI, DIIDW, KJD, MEDLINE, RSCI, SCIELO, ZOOREC Timespan=All years Search language=Auto*
#3	251,883	TS=(COVID-19 OR Coronavirus OR SARS-CoV-2 OR post-COVID OR long-COVID OR long-term-COVID)<br>*Databases= WOS, BCI, BIOSIS, CCC, DRCI, DIIDW, KJD, MEDLINE, RSCI, SCIELO, ZOOREC Timespan=All years Search language=Auto*
#2	55	TS=(post-covid NEAR Fatigue)<br>*Databases= WOS, BCI, BIOSIS, CCC, DRCI, DIIDW, KJD, MEDLINE, RSCI, SCIELO, ZOOREC Timespan=All years Search language=Auto*
#1	206,694	TOPIC: (covid-19)<br>*Databases= WOS, BCI, BIOSIS, CCC, DRCI, DIIDW, KJD, MEDLINE, RSCI, SCIELO, ZOOREC Timespan=All years Search language=Auto*

PsycInfo

SearchID	Search terms	Results
S1	AB coronavirus OR AB covid-19 OR AB sars-cov-2	6,820
S2	AB long term OR AB post viral fatigue OR AB post viral fatigue syndrome OR AB post infection OR chronic fatigue OR chronic fatigue syndrome	148,420
S3	S1 AND S2	412 (236)
